# Sensory feedback in Parkinson's disease patients with “on”-predominant freezing of gait

**DOI:** 10.3389/fneur.2013.00014

**Published:** 2013-02-25

**Authors:** Alberto J. Espay, Laura Gaines, Rahul Gupta

**Affiliations:** ^1^Department of Neurology, Gardner Family Center for Parkinson's Disease and Movement Disorders, University of Cincinnati School of MedicineCincinnati, OH, USA; ^2^Neuromodulation Research, Medtronic NeuromodulationMinneapolis, MN, USA

Freezing of gait (FOG) often occurs during the course of Parkinson's disease (PD), generating substantial disability (Giladi et al., 2001). Auditory, visual or tactile stimuli have been shown to improve FOG in PD and cueing-assisted modifications of gait have been proposed (Thaut et al., 1996) including a closed-loop augmented-reality device we reported for use in patients with “off”-related FOG (Baram et al., 2002; Espay et al., 2010). The two main limitations of prior studies were (1) the focus on “off”-only FOG, which arguably could have also been improved by pharmacotherapeutic strategies aimed at minimizing “off” time, and (2) the uncertainty of long-term residual benefits. We therefore sought to evaluate the immediate and longer-term effects of a one-month at-home training with the previously reported closed-loop augmented-reality cueing device on PD patients with predominantly “on” FOG.

## Patients and methods

**Figure 1:**
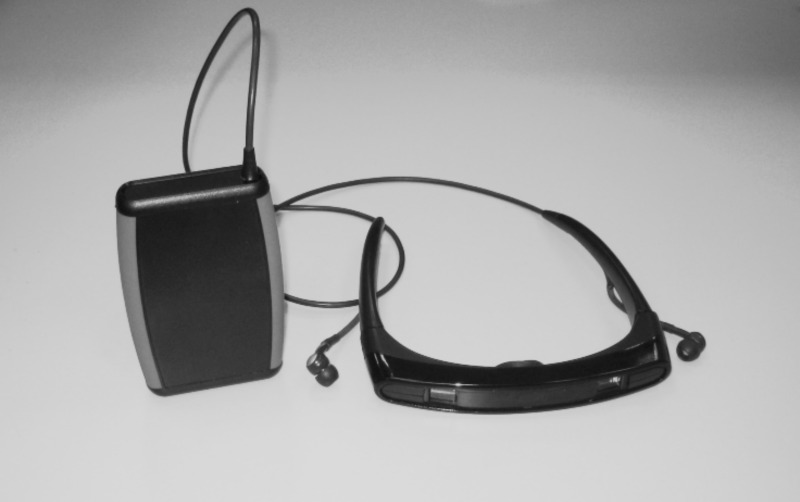
**Virtual reality goggles used in this study, containing a built-in LCD screen between the visors which projects floor tiles when subjects are moving, and earphones sounding a step-matched cue as determined by the connected sensor, strapped at the belt**.

We recruited 13 consecutive consenting PD patients with mild to moderate gait impairment and “on”-predominant FOG (7 men, 6 women; mean age: 69.1 ± 18 years, UPDRS-III: 28.5 ± 4.5 [all subjects were tremorless]; disease duration: 13.6 ± 14 years; levodopa dose: 788 ± 412 mg/day; FOGQ: 15.2 ± 3.5); scoring >1 on the motor subscale of the Unified Parkinson's disease rating scale (UPDRS-III) item 29, on intermittent but not continuous ambulatory aid, and Mini-Mental State score of ≥25. Three patients treated with STN DBS with similar gait impairment were also included. We excluded patients with associated musculoskeletal disorders such as severe arthritis, post-knee surgery, hip surgery or any other condition that independently impaired gait. The study followed a single-blinded prospective cohort design. The device is composed of a small measurement-computation unit attached to the patient's clothing, a head-mounted microdisplay, and earphones, and contains a multiaxial accelerometer, a compass, and a microcontroller (Figure 1). The apparatus, operating in an adaptive closed-loop mode, displays a life-size virtual checkerboard-tiled floor superimposed on the real world with specialized see-through glasses. The user regulates the gait pattern to create a constant optical flow and a rhythmic auditory cue. Additional technical aspects have been previously described (Espay et al., 2010). All subjects underwent a standardized preliminary assessment, including UPDRS-III and self-administered Freezing of Gait Questionnaire (FOGQ) (Giladi et al., 2000) to ascertain their motor and gait-specific function both before and after one-month training with the augmented-reality device described previously (Espay et al., 2010). All assessments took place without the device in the “on” state at baseline, after 4 weeks of at home-training, and monthly after discontinuing device use for up to 16 weeks (residual effect). The University of Cincinnati Institutional Review Board approved this study receive and written informed patient consent was obtained from all subjects.

## Results

Severity of disease and associated frailty in the target population greatly limited recruitment and completion of this 4-month study, forcing its early termination after 2.5 years. During that period, from a total of 16 eligible patients, 13 were enrolled but only 2 completed the study. The high dropout rate was mostly due to the severe burden of disability and advanced disease of “on” freezers, limiting the opportunities to fulfill the required daily training sessions and preventing their return for the scheduled study visits. We documented a single responder: a 62-year-old woman with 15-year disease duration (levodopa dose: 600 mg/day; UPDRS-III: 24 at baseline, 15.5 at 12 weeks; FOGQ: 16 at baseline, 13 at 12 weeks). Benefits were renewed after a “booster” training once the residual gait improvement weaned at about 16 weeks post-training.

## Discussion

“On”-predominant FOG remains refractory to many interventions compared with “off”-only PD freezers. Our findings suggest that training with sensory feedback may not be a viable therapy alternative for the majority of patients with “on”-predominant FOG, mainly due to the advanced disease state and the associated frailty of the population (Kompoliti et al., 2000). At this advanced disease stage, the development of various comorbidities, including dementia, also limit consideration of deep brain stimulation (Fasano et al., 2010). It is unknown whether the earlier application of sensory feedback-focused exercise programs, such as the augmented-reality device used here, may delay the onset of this disabling and intractable motor milestone of PD. Alternative interventions, such as riding a bicycle, may yield greater benefit in this highly disabled population (Snijders et al., 2011).

## Funding

This study was funded by Medtronic Neuromodulation through an investigator-initiated research application. The sponsor had no role in data collection or manuscript drafting.

## References

[B1] Baram Y. Aharon-Peretz J. Simionovici Y. Ron L. (2002). Walking on virtual tiles: virtual reality in closed loop improves gain in Parkinson's patients. Neural Proc. Lett. 16, 227–233

[B2] Espay A. J. Baram Y. Dwivedi A. K. Shukla R. Gartner M. Gaines L. (2010). At-home training with closed-loop augmented-reality cueing device for improving gait in patients with Parkinson disease. J. Rehabil. Res. Dev. 47, 573–581 20848370 10.1682/jrrd.2009.10.0165

[B3] Fasano A. Romito L. M. Daniele A. Piano C. Zinno M. Bentivoglio A. R. (2010). Motor and cognitive outcome in patients with Parkinson's disease 8 years after subthalamic implants. Brain 133, 2664–2676 10.1093/brain/awq221 20802207

[B4] Giladi N. McDermott M. P. Fahn S. Przedborski S. Jankovic J. Stern M. (2001). Freezing of gait in PD: prospective assessment in the DATATOP cohort. Neurology 56, 1712–1721 11425939 10.1212/wnl.56.12.1712

[B5] Giladi N. Shabtai H. Simon E. S. Biran S. Tal J. Korczyn A. D. (2000). Construction of freezing of gait questionnaire for patients with Parkinsonism. Parkinsonism Relat. Disord. 6, 165–170 10817956 10.1016/s1353-8020(99)00062-0

[B6] Kompoliti K. Goetz C. G. Leurgans S. Morrissey M. Siegel I. M. (2000). “On” freezing in Parkinson's disease: resistance to visual cue walking devices. Mov. Disord. 15, 309–312 10752582 10.1002/1531-8257(200003)15:2<309::aid-mds1016>3.0.co;2-p

[B7] Snijders A. H. Toni I. Ruzicka E. Bloem B. R. (2011). Bicycling breaks the ice for freezers of gait. Mov. Disord. 26, 367–371 10.1002/mds.23530 21462254

[B8] Thaut M. H. McIntosh G. C. Rice R. R. Miller R. A. Rathbun J. Brault J. M. (1996). Rhythmic auditory stimulation in gait training for Parkinson's disease patients. Mov. Disord. 11, 193–200 10.1002/mds.870110213 8684391

